# Case Report: Focal leptomeningeal disease, atypical cancer of unknown primary site

**DOI:** 10.12688/f1000research.122434.2

**Published:** 2022-09-21

**Authors:** Miguel A. Vences, Mary M. Araujo-Chumacero, Diego Urrunaga-Pastor, Leila Barreto, Liliana Rodríguez-Kadota, Elliot Barreto-Acevedo, César Saavedra-Rocha, Elder V. Quispe-Huamaní

**Affiliations:** 1 Departamento de Neurología, Hospital Nacional Edgardo Rebagliati Martins, EsSalud, Lima, Peru; 2 Unidad para la Generación y Síntesis de Evidencias en Salud, Universidad San Ignacio de Loyola, Lima, Peru; 3 Dirección de Investigación en Salud, Instituto de Evaluación de Tecnologías en Salud e Investigación – IETSI, EsSalud, Lima, Peru

**Keywords:** Dementia, Brain metastasis, Nervous System Neoplasms, PET-CT scan, Leptomeningeal Neoplasms

## Abstract

**Background:** Leptomeningeal metastasis is an infrequent form of cancer expression, and it has a poor prognosis due to its torpid evolution and its challenging diagnosis.

**Case report: **We report the case of a 68-year-old woman with rapidly progressing cognitive decline and focal epilepsy. Brain magnetic resonance imaging showed extensive gyriform hypersignal in the right precentral sulcus region, without mass effect, tenuous contrast uptake, and hydrocephalus with transependymal edema. The body tomographic study was negative for solid cancer and the 18F-FDG PET-CT revealed a severe hypermetabolism in the right lung upper lobe. These findings were suggestive of lung cancer with leptomeningeal metastasis. We performed a brain biopsy, finding atypical cells in the leptomeningeal region with positive immunohistochemical staining for CK7 and negative for CK20 corresponding to lung adenocarcinoma. The patient was evaluated in the oncology service and scheduled for radiotherapy and chemotherapy.

**Conclusions:** Focal leptomeningeal disease is an entity that should be considered as a differential diagnosis in all cases of focal leptomeningitis. Timely diagnosis and adequate cancer management can increase patient survival.

## Introduction

Leptomeningeal metastasis is an infrequent form of cancer expression, and its association has been described with solid cancer such as breast, lung and melanoma. ^1^ This entity has a poor prognosis due to its torpid evolution and its diagnosis is a challenge, since on certain occasions it is the first systemic manifestation of cancer not yet diagnosed. ^2^

We present the case of a 68-year-old woman with leptomeningeal metastasis as the first systemic manifestation of an unknown primary origin cancer. In addition, we carried out a review of the literature.

## Case report

We describe the case of a 68-year-old self-sufficient housewife and Latin woman, with no significant family health history, diagnosed of hypothyroidism since she was young and who suffered from COVID-19 pneumonia with no severity criteria four months before admission. She was admitted to the neurology service due to a clinical course of insidious onset and progressive course of 4 months, characterized by cognitive decline and change in her behavior with affective flattening. In the seven days prior to admission, she developed mutism and an episode of tonic-clonic epileptic seizure of unknown onset and self-limited with a postictal dystonic posture in the left upper limb lasting less than one minute. She did not receive treatment for these symptoms prior to admission.

The neurological examination showed an apathy-abulic patient, person-oriented, with significantly impaired working memory, language without fluency with null judgment and introspection. In addition, she had mild left brachio-crural motor deficit. The rest of the physical examination showed no alterations.

In the neuropsychological assessment, we found cognitive impairment evidenced by a low score in the Folstein Minimental State Examination (MMSE) ^3^ and the Montreal Cognitive Assessment (MoCA), ^4^ with 9 points and 12 points, respectively.

**Figure 1.**
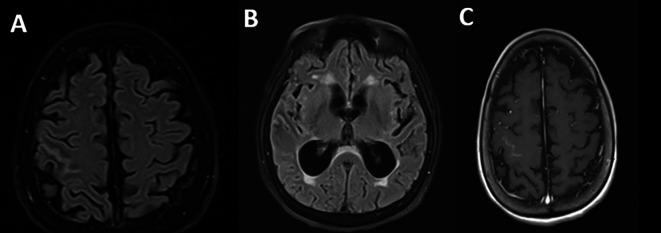
Brain magnetic resonance imaging: A. Irregular leptomeningeal thickening at the right Rolando fissure (thickness 2 mm), as well as smaller adjacent focal parietal leptomeningeal thickening (FLAIR sequence); B. Supratentorial hydrocephalus of recent onset associated with interstitial/transependymal edema (FLAIR sequence); C. No expansive lesions or enhancements in the brain parenchyma (T1 with contrast).

Regarding the imaging studies performed, brain tomography without contrast did not show alterations and brain magnetic resonance imaging showed extensive leptomeningeal gyriform hypersignal in the right precentral sulcus region without mass effect and tenuous contrast uptake, associated with hydrocephalus and transependymal edema ( Figure 1).

Due to the leptomeningeal gyriform finding in the brain magnetic resonance imaging and the patient’s clinical course, laboratory tests and cerebrospinal fluid examination were requested. They reported hyperproteinorrhachia (149.4 milligrams per deciliter) without glucose consumption or pleocytosis. The rest of the studies were negative for inflammatory disease (oligoclonal bands in serum and cerebrospinal fluid), autoimmune (C3, C4, antinuclear antibodies, antineutrophil cytoplasmic antibodies, extractable nuclear antigens and rheumatoid factor), neoplastic (cerebrospinal fluid Papanicolau smear, cytometry and immunofixation in cerebrospinal fluid) and associated infectious (serological test for syphilis; antibody serological test for toxoplasmosis, rubella, cytomegalovirus, herpes simplex; cytomegalovirus viral load; John Cunningham virus in serum and cerebrospinal fluid; herpes virus in cerebrospinal fluid; HIV ELISA; Epstein-Barr virus in serum and cerebrospinal fluid; human T-lymphotropic virus type I and II; adenosine deaminase in cerebrospinal fluid and Koch’s bacillus in sputum, urine, gastric juice, feces and cerebrospinal fluid). Tumor markers as CA 125, CA 15-3 and CYFRA 21.1 were negative, excepting carcinoembryonic antigen (CEA) and alpha fetoprotein (AFP) at levels of 246.24 ng/mL and 12.63 ng/mL, respectively. Contrast-enhanced cervical, thoracic, abdominal, and pelvic tomography excluded the presence of solid neoplasms.

Based on these results, the case was approached as a leptomeningeal metastasis of unknown primary origin, probably from the gastrointestinal tract. For this reason, they extended the study with a fecal occult blood test, which was positive on two opportunities, and performed endoscopic studies twice (upper digestive endoscopy and colonoscopy), with no evidence of cancer in the gastrointestinal tract studied. The gastroenterologists suggested expanding the study with high-resolution enterography to investigate a possible cancer lesion in the midgut. They performed it, without obtaining a positive result.

**Figure 2.**
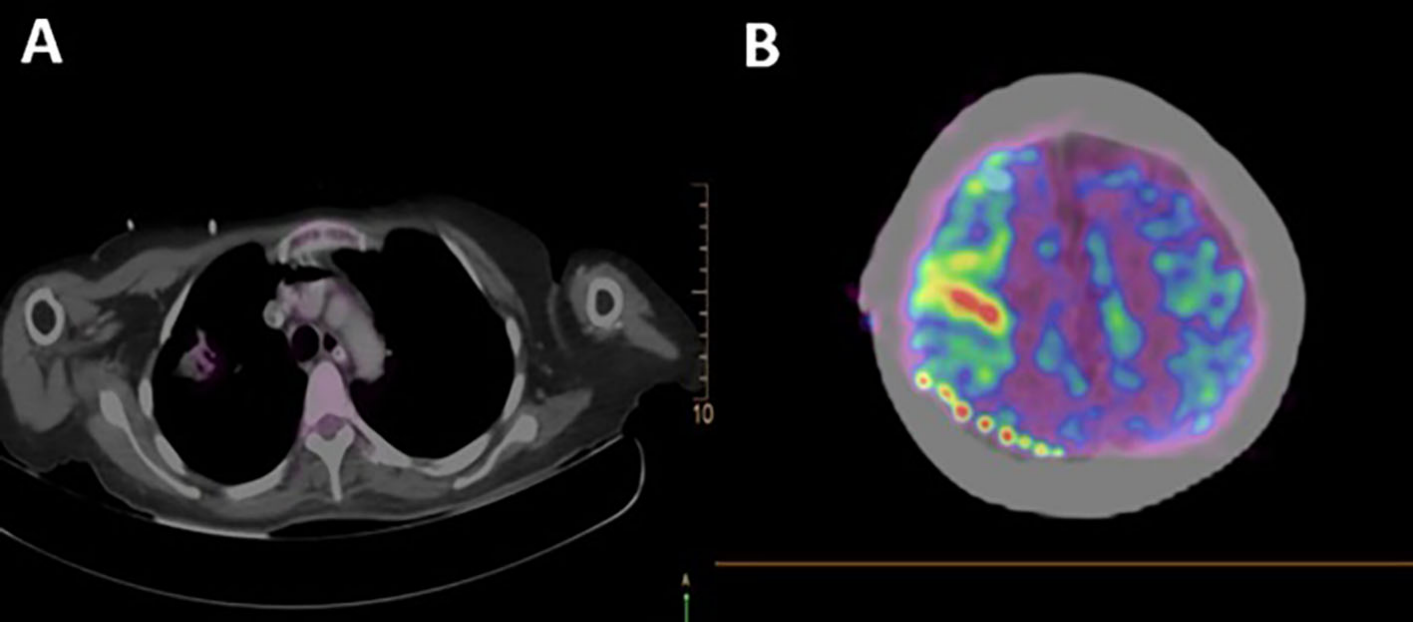
Total body 18F-fluorodeoxyglucose positron emission tomography (18F-FDG PET-CT): A. Pulmonary hypermetabolism without neoplastic mass in tomographic scanning (lung); B. FDG hypermetabolism at the right parietal region (cerebral).

A total body 18F-fluorodeoxyglucose positron emission tomography (18F-FDG PET-CT) was performed on the patient with a GEMINI TF64 equipment in helical mode. It revealed severe hypermetabolism in the anterior segment of the right lung upper lobe (SUVmax: 5.47) and at the right precentral cerebral sulcus. These findings were suggestive of pulmonary neoplasia with leptomeningeal metastases ( Figure 2).

**Figure 3.**
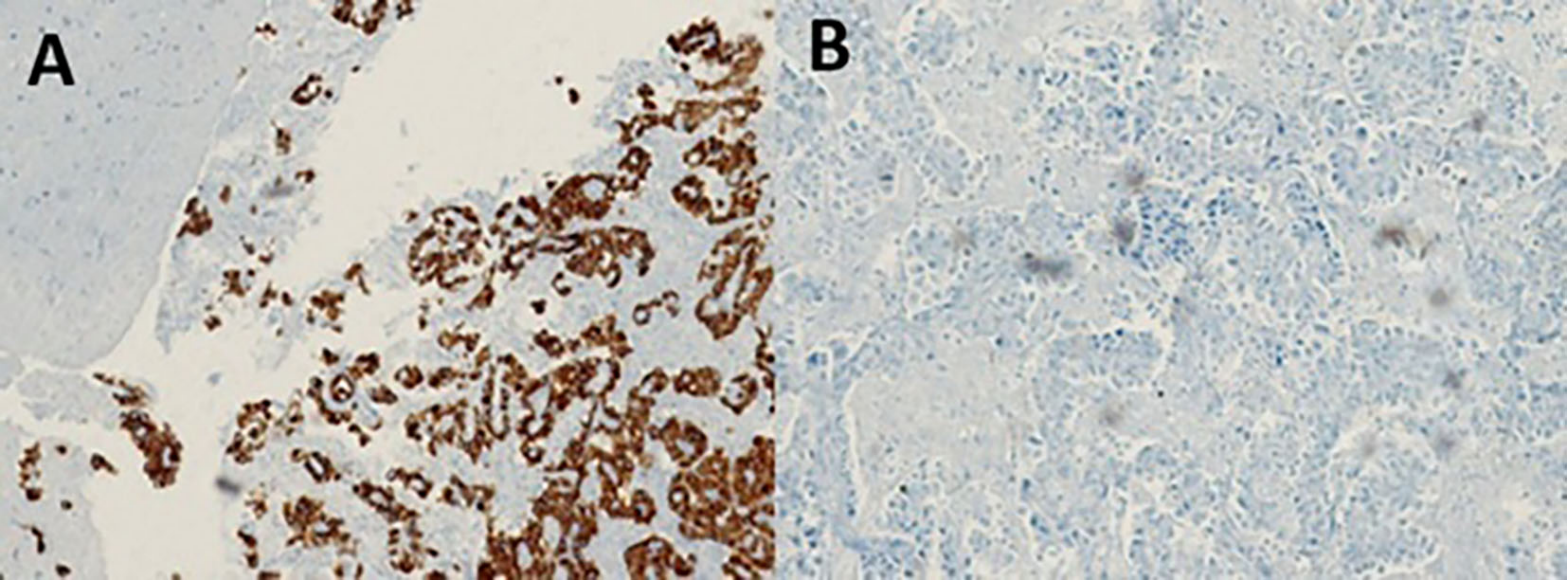
Pathology slides with immunohistochemistry and markers used in unknown primary adenocarcinoma and corresponding to lung adenocarcinoma: A. Atypical cells in the leptomeningeal region with positive staining for CK7; B. Atypical cells in the leptomeningeal region with negative staining for CK20.

With the suggestive result of the PET-CT that the focus of the primary cancer was pulmonary, the patient underwent bronchoalveolar lavage with a transbronchial biopsy without a positive result. For this reason, we decided to perform a stereotactic-guided brain biopsy, whose pathology result was inconclusive, and the pathology service requested a new sample. The patient underwent a new intervention with an open brain biopsy, obtaining samples from the right leptomeningeal region, confirming the diagnosis of poorly differentiated primary non-small cell lung adenocarcinoma ( Figure 3). We performed a ventriculoperitoneal shunt due to the hydrocephalus that she presented.

The patient was evaluated by the oncology service, where they applied the Karnofsky Performance State (KPS) scale, ^5^ in which the patient obtained 40 points. Then, they opted for a therapeutic plan of radiotherapy and subsequent chemotherapy.

Three months after diagnosis, she has completed 10 sessions of brain radiotherapy with no adverse events and is undergoing outpatient chemotherapy. The patient’s family reports an improvement in the attention and language level, with better fluency. Neuropsychological tests showed an improvement of the cognitive disorder (MMSE: 15 and MoCA: 19).

## Discussion

Classic leptomeningeal metastasis is the result of the infiltration of cancer cells in the meninges. ^6^ Breast cancer, melanoma, and lung cancer, which occurs in up to 5% of cases, are the neoplasms with greater trophism for the meninges. ^7^ This pathology does not require a biopsy for its diagnosis and the classification according to the European Society of Neuro-oncology is based on the cerebrospinal fluid study and the magnetic resonance images. However, performing a biopsy is recommended when there is no previously demonstrated solid cancer, since the oncological treatment will depend on this. ^8^

In this report, we describe a difficult diagnosis case, with a particular pattern of leptomeningeal metastasis of unknown primary origin called focal leptomeningeal disease. ^9^ Previous reports have described this entity in non-small cell lung cancer patients. They found in them a higher survival compared to classic leptomeningeal disease patients after a targeted treatment specific to the cancer cells mutation. ^10^^,^^11^

Regarding the physiopathology of this disease, it has been postulated that the perivascular spaces with the perivascular channels act as a bridge through angiotropic mechanisms. ^9^^,^^12^^,^^13^ In addition, the association of dural thickening and enhancement has been reported, as a radiological expression of tumor involvement and venous congestion secondary to the perivascular involvement of the disease. ^9^^,^^11^^,^^14^

The neurological symptoms associated with this condition have been described as less severe than those associated with classic leptomeningeal disease. The most frequent symptoms in focal leptomeningeal disease were motor deficit, headache, and cognitive impairment. ^9^^,^^15^ In our patient, we also reported a self-limited focal onset epileptic seizure, being consistent with the right parietal structural lesion.

The cases described by Dasgupta *et al.* have the same radiological pattern of focal leptomeningeal enhancement in patients with epidermal growth factor receptor-mutated non-small cell lung cancer treated with tyrosine kinase inhibitors and radiation therapy. ^9^ However, our case is atypical because solid lung cancer was not demonstrated as in the previous case. Then, we highlight PET-CT as a highly supportive tool for diagnosis in cancer of unknown primary origin. ^16^

We do not consider COVID-19 history as an important element for the onset of temporal cognitive impairment. Our patient presented additional clinical manifestations that do not correspond to this association, ^17^ as mutism, focal neurological deficit, and epileptic seizure. This could be confirmed after observing the leptomeningeal hypersignal in the MRI, then, we extended the study searching for other etiologies.

The main strength of our case report was the access to important diagnostic tests, including PET-CT and immunohistochemistry, which allowed us to identify the final diagnosis despite the atypical manifestation of the disease. However, the main limitation was the limited follow-up time (no more than three months), thus, we were unable to perform more clinical and radiological controls.

In conclusion, this is an atypical case of leptomeningeal metastasis in the absence of solid cancer in the tomography, demonstrated with the support of PET-CT and histologically confirmed by leptomeningeal biopsy. Focal leptomeningeal disease is an entity that should be considered as a differential diagnosis in focal leptomeningitis cases with rapidly cognitive impairment progression. Timely diagnosis and adequate oncological management can increase patients’ survival.

### Ethical approval and informed consent

This article has been approved by the institutional ethics committee of the Red Prestacional Rebagliati (REGISTER 086-2021). We obtained the patient’s written informed consent to participate in this study and for the publication of images and data included in this case report.

## Authors’ contributions

MAV, MMAC, LB, LRK, EBA, CSR, EVQH treated the patient, interpreted the neuroradiologic imaging and laboratory markers. All authors participated in design, writing, critical review and approved the final version of the manuscript.

## Data availability

All data underlying the results are available as part of the article and no additional source data are required.
