# Supplementary material for: Case Report: Focal leptomeningeal disease, atypical cancer of unknown primary site
Source: F1000Res. 2022 Sep 21;11:885. Originally published 2022 Aug 2. [Version 2] doi: 10.12688/f1000research.122434.2 (PMC9508522; doi:10.12688/f1000research.122434.2)
Supplement: Peer review history

# Peer review: Reviewer response for version 2

**DOI:** 10.5256/f1000research.138679.r151090

**Published:** 2022-09-23

**Authors:** José Percy Amado Tineo

**Affiliations:** 1 Facultad de Medicina Humana, Universidad Nacional Mayor de San Marcos, Lima, Peru

I have reviewed the answers of the authors and they seem adequate to me. I have no further observations.

Are enough details provided of any physical examination and diagnostic tests, treatment given and outcomes?

Yes

Is the case presented with sufficient detail to be useful for other practitioners?

Yes

Is sufficient discussion included of the importance of the findings and their relevance to future understanding of disease processes, diagnosis or treatment?

Yes

Is the background of the case’s history and progression described in sufficient detail?

Yes

Reviewer Expertise:

Internal medicine

I confirm that I have read this submission and believe that I have an appropriate level of expertise to confirm that it is of an acceptable scientific standard.

# Peer review: Reviewer response for version 1

**DOI:** 10.5256/f1000research.134420.r146382

**Published:** 2022-09-06

**Authors:** José Percy Amado Tineo

**Affiliations:** 1 Facultad de Medicina Humana, Universidad Nacional Mayor de San Marcos, Lima, Peru

I appreciate the opportunity to review this case report, I find it very interesting and well written.

 I have some observations that I will mention:

- I do not think it is necessary to include the name of the country in the title, since the place of origin is not relevant in this case.
- It would be necessary to include a comment on the association between COVID-19 and cognitive impairment, giving more detail to this history in the patient, since it has a temporal relationship with the onset of cognitive impairment (4 months)
- Although it is true that the PET-SCAN shows lesions suggestive of lung cancer, this could not be confirmed by pathological anatomy, can we comment on the possibility that the origin of the adenocarcinoma found in the meninges is from another organ?
- It would be appropriate to know the final destination of the patient, if chemotherapy was completed after radiotherapy and her survival, considering that having distant metastases would be a clinical stage IV with low short-term survival.
- When writing bibliographical references, the first 6 authors are usually mentioned and in several references only 3 are mentioned.

Are enough details provided of any physical examination and diagnostic tests, treatment given and outcomes?

Yes

Is the case presented with sufficient detail to be useful for other practitioners?

Yes

Is sufficient discussion included of the importance of the findings and their relevance to future understanding of disease processes, diagnosis or treatment?

Yes

Is the background of the case’s history and progression described in sufficient detail?

Yes

Reviewer Expertise:

Internal medicine

I confirm that I have read this submission and believe that I have an appropriate level of expertise to confirm that it is of an acceptable scientific standard, however I have significant reservations, as outlined above.

# Response

**Published:** 2022-09-14

**Authors:** Diego Urrunaga-Pastor

**Affiliations:** USIL, Peru

Thanks for the comments. We answered to comments in detail.

 1. I do not think it is necessary to include the name of the country in the title, since the place of origin is not relevant in this case.

**Answer**: Thanks for the comment. We have deleted the country of origin from the title case.

 2. It would be necessary to include a comment on the association between COVID-19 and cognitive impairment, giving more detail to this history in the patient, since it has a temporal relationship with the onset of cognitive impairment (4 months).

**Answer**: Thanks for the comment. We added in first paragraph from case report section that our patient COVID-19 pneumonia did not have severity criteria. In addition, we do not consider COVID-19 history as an important element for the onset of temporal cognitive impairment because the patient presented additional clinical manifestations that do not correspond to this association (1), as mutism, focal neurological deficit, and epileptic seizure. This could be confirmed after observing the leptomeningeal hypersignal in the MRI, then, we extended the study searching for other etiologies. We added a paragraph in discussion about this topic.

 3. Although it is true that the PET-SCAN shows lesions suggestive of lung cancer, this could not be confirmed by pathological anatomy, can we comment on the possibility that the origin of the adenocarcinoma found in the meninges is from another organ?

**Answer**: Thanks for the comment. We obtained samples from the right leptomeningeal region through a brain biopsy, confirming the diagnosis of poorly differentiated primary non-small cell lung adenocarcinoma (Figure 3). We performed a ventriculoperitoneal shunt due to the hydrocephalus that she presented. This was described in the case report.

 4. It would be appropriate to know the final destination of the patient, if chemotherapy was completed after radiotherapy and her survival, considering that having distant metastases would be a clinical stage IV with low short-term survival.

**Answer**: Thanks for the comment. We agree with the importance of follow-up in this patient, however, we could only follow-up her to 3 months. Then, we described it as a limitation.

 5. When writing bibliographical references, the first 6 authors are usually mentioned and in several references only 3 are mentioned.

**Answer**: Thanks for the comment. We reviewed the references and most of them had fewer than 6 authors, so we did not mention et al.

**References**

 1.           Mattioli F, Stampatori C, Righetti F, Sala E, Tomasi C, De Palma G. Neurological and cognitive sequelae of Covid-19: a four month follow-up. Journal of neurology. 2021;268(12):4422-8.

# Peer review: Reviewer response for version 1

**DOI:** 10.5256/f1000research.134420.r146386

**Published:** 2022-08-23

**Authors:** Miguel Oswaldo Cadena-Sanabria

**Affiliations:** 1 Internal Medicine Department, Industrial University of Santander, University Hospital of Santander, Bucaramanga, Colombia; 2 Universidad Autónoma de Bucaramanga, Bucaramanga, Colombia

This case highlights the importance of a standardized and progressive approach to elderly people with rapidly progressive cognitive impairment, whose etiologies include paraneoplastic or metastatic lesions. To highlight the gradual development of microbiological, cytological and imaging diagnostic aids. The PET Scan is becoming more and more important in the determination of hypermetabolic lesions that guide towards a possible tumor lesion. It is also important to discourage studies with tumor markers, since in this case they only generated confusion of a possible primary at the gastrointestinal level. Since it is lung adenocarcinoma, it is important to document the presence of mutated epidermal growth factor receptor [EGFR] and anaplastic lymphoma kinase [ALK] translocation due to its implications for immunotherapy and prognosis, as well as thromboembolic risk. Finally, being an older woman, I share a reference about the need to carry out an approach guided by her comprehensive geriatric assessment. Here the authors share with us the evolution in neuropsychological tests, evidencing the positive impact of radiotherapy intervention.

Are enough details provided of any physical examination and diagnostic tests, treatment given and outcomes?

Yes

Is the case presented with sufficient detail to be useful for other practitioners?

Yes

Is sufficient discussion included of the importance of the findings and their relevance to future understanding of disease processes, diagnosis or treatment?

Yes

Is the background of the case’s history and progression described in sufficient detail?

Yes

Reviewer Expertise:

Geriatric assessment, frailty, cognitive disorders, onco-geriatrics

I confirm that I have read this submission and believe that I have an appropriate level of expertise to confirm that it is of an acceptable scientific standard.

## References

[1] : Importancia de la valoración geriátrica integral en el manejo de pacientes con cáncer. *Médicas UIS* .25(2) : Reference source
